# A meta-analysis of medicinal plants to assess the evidence for toxicity

**DOI:** 10.2478/v10102-010-0016-0

**Published:** 2010-06

**Authors:** Sarah Chen, Amandio Vieira

**Affiliations:** Nutrition and Metabolic Research Laboratory, Kines-9600, Simon Fraser University, Burnaby, B.C., Canada

**Keywords:** phytotoxicity, nephrotoxicity, hepatotoxicity, phytomedicinal informatics, *Aristolochia*, *Larrea*

## Abstract

Toxicity of phytochemicals, plant-based extracts and dietary supplements, and medicinal plants in general, is of medical importance and must be considered in phytotherapy and other plant uses. We show in this report how general database analyses can provide a quantitative assessment of research and evidence related to toxicity of medicinal plants or specific phytochemicals. As examples, several medicinal plants are analyzed for their relation to nephrotoxicity and hepatotoxicity. The results of analyses in different databases are similar, and reveal the two best-established toxic effects among the group of plants that were examined: nephrotoxicity of *Aristolochia fangchi* and hepatotoxicity of *Larrea tridentata*.

## Introduction

Phytotoxicity is an important concern in phytomedicine and other situations where potentially toxic plants are ingested. A clinician or researcher may want to obtain a quantitative indication of the best-established or most-researched phytotoxic effect of a given medicinal plant (cf. Suter *et al*., 2004; Kiefer *et al*., 2001). To provide a large-scale database analyses in such a context, a phytomedicinal informatics approach is described. As examples, six medicinal plants – *Allium sativum (A.s.); Echinacea purpurea (E.p.); Mormordica charantia (M.c.); Aristolochia fangchi (A.f.); Larrea tridentate (L.t.); Zingiber officinalis (Z.o.); Vaccinium myrtillus (V.m.)* – are analyzed for parameters related to nephrotoxicity (Nx) and hepatotoxicity (Hx).

To better illustrate the method and provide a larger base for comparisons, other parameters related to acute and chronic medical conditions are also included: nausea (Na), diabetes (Db), and inflammation (If). Some of the plants were selected because of well-documented specific therapeutic associations, *e.g.*, *Momordica* and potential applications to diabetes (Chatterjee, 1963; Leatherdale *et al*., 1981; Yadav *et al*., 2005; Jung *et al*., 2006). *Aristolochia* and *Larrea* were selected because of their known toxicities. *Aristolochia* and its constituent aristolochic acids have reported nephrotoxicity, and their use may increase the risk urothelial carcinomas (Debelle *et al*., 2002; Debelle *et al*., 2002; Ioset *et al*., 2003; Martinez *et al*., 2002; Yuan *et al*., 2009). For *Larrea* and one of its polyphenol components, nordihydroguaiaretic acid, hepatotoxicity and pro-oxidative activity have been reported (Gay and Musser, 2008; Sahu *et al*., 2006). These plants could, thus, represent internal controls for the exemplary analyses reported in this work and for future analyses of other plants, toxicity parameters, or databases. Some plants commonly used in phytotherapy (cf. Ness, 1999) with a wide range of reported therapeutic effects were also included for comparison, *e.g.*, *Echinacea* and *Allium*.

Quantitative determinations of toxicity-plant associations, referred to as an association index (Ai, see Methods section below), can be used to compare different databases and provide a measure of the best-established phytotoxic effects of a given medicinal plant, or identify the plant with the most evidence for a particular toxic effect such as hepatotoxicity. As an example of the method, two types of databases were used. The first database searched was Medline (PubMed), a health-specific database: *http://www.ncbi.nlm.nih.gov/entrez/query.fcgi?DB=pubmed*. The second database searched was the internet (world-wide web, www; searched with the Google™ search engine *http://www.google.ca/*), a general database that is not limited to scientific evidence (see Results and Discussion section).

## Methods

The first step in the analysis is to determine the number of identified database cases (‘hits’) for a given plant (Pl) such as ‘*Aristolochia fangchi’*, and for a given biomedical parameter (Bm) such as ‘nephrotoxicity’. The second step is to determine the number of identified database cases for the combination (Co) of the above two parameters, one plant and one biomedical, *e.g.*, number of cases or hits for ‘*Aristolochia fangchi* and nephrotoxicity’. The corrected association index (Ai) of the two parameters is then determined by the following calculation: Ai = (Co/Bm) (Co/Pl).

Dividing the Co term by each of its components individually (Bm or Pl), as shown in the above equation, provides an important abundance correction, and allows for more accurate Ai estimates when comparing extensively researched plants or toxicities with those plants and toxic activities that are much less extensively researched.

**Figure 1:**
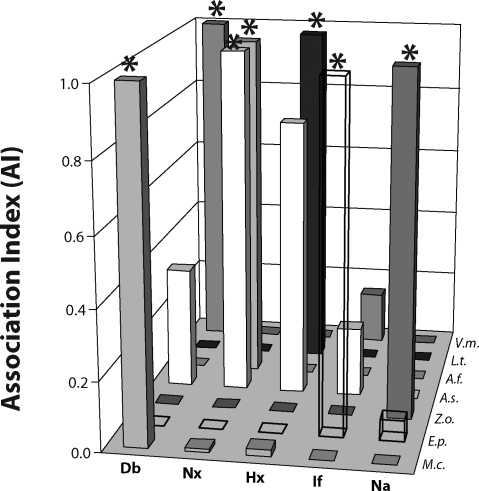
Relative association indices for each plant with the five biomedical terms. A value of 1 indicates the one, strongest biomedical association for that particular plant (asterisk). This graph is based on Medline PubMed data. The ‘AI’ of the y-axis represents rAi (see text for its definition).

The largest Ai value for a given plant (*e.g.*, the Ai for *M.c.*-Db in Figure 1) is used to calculate all the relative Ai values (rAi) for that same plant with each of the biomedical terms. For example, the calculation of all the rAi values for the plant *M.c.* was performed using the following five equations (the bars that correspond to these values can be seen in Figure 1 along the *M.c.* axis):

$$\text{rA}{\text{i}}_{\text{McDb}}\text{=}\left(\text{A}{\text{i}}_{\text{McDb}}\right)\text{/}\left(\text{A}{\text{i}}_{\text{McDb}}\right)\text{=1}$$

$$\text{rA}{\text{i}}_{\text{McNb}}\text{=}\left(\text{A}{\text{i}}_{\text{McNa}}\right)\text{/}\left(\text{A}{\text{i}}_{\text{McDb}}\right)\ll \text{1}$$

$$\text{rA}{\text{i}}_{\text{McHx}}\text{=}\left(\text{A}{\text{i}}_{\text{McHx}}\right)\text{/}\left(\text{A}{\text{i}}_{\text{McDb}}\right)\ll \text{1}$$

$$\text{rA}{\text{i}}_{\text{McNx}}\text{=}\left(\text{A}{\text{i}}_{\text{McNx}}\right)\text{/}\left(\text{A}{\text{i}}_{\text{McDb}}\right)\ll \text{1}$$

$$\text{rA}{\text{i}}_{\text{Mclf}}\text{=}\left(\text{A}{\text{i}}_{\text{Mclf}}\right)\text{/}\left(\text{A}{\text{i}}_{\text{McDb}}\right)\ll \text{1}$$

**Figure 2:**
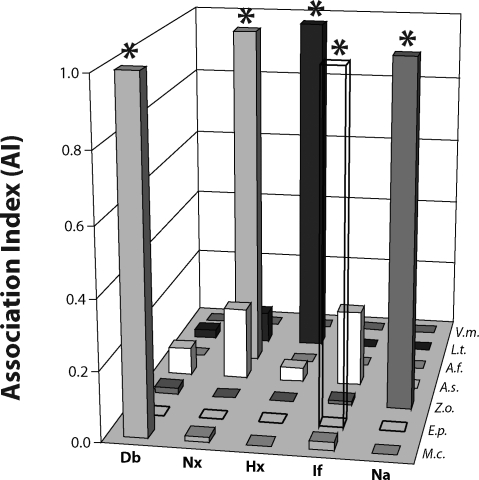
Relative association indices for each biomedical term with the seven plants. A value of 1 indicates the one, strongest plant association for that particular biomedical term (asterisk). This graph is based on Medline PubMed data. The ‘AI’ of the y-axis represents rAi (see text for its definition).

Similarly, the largest Ai value for a given biomedical parameter (*e.g.*, the Ai for Nx-*A.f.* in Figure 2) is used to calculate all the relative Ai values (rAi) for that same biomedical term with each of the plants. For example, the calculation of all the rAi values for the Nx (nephrotoxicity) term was performed using the following seven equations (the bars that correspond to these values can be seen in Figure 2 along the Nx axis):

$$\text{rA}{\text{i}}_{\text{NxAf}}\text{=}\left(\text{A}{\text{i}}_{\text{NxAf}}\right)\text{/}\left(\text{A}{\text{i}}_{\text{NxAf}}\right)\text{=1}$$

$$\text{rA}{\text{i}}_{\text{NxMc}}\text{=}\left(\text{A}{\text{i}}_{\text{NxMc}}\right)\text{/}\left(\text{A}{\text{i}}_{\text{NxAf}}\right)\ll \text{1}$$

$$\text{rA}{\text{i}}_{\text{NxAs}}\text{=}\left(\text{A}{\text{i}}_{\text{NxAs}}\right)\text{/}\left(\text{A}{\text{i}}_{\text{NxAf}}\right)\text{<1}$$

$$\text{rA}{\text{i}}_{\text{NxEp}}\text{=}\left(\text{A}{\text{i}}_{\text{NxEp}}\right)\text{/}\left(\text{A}{\text{i}}_{\text{NxAf}}\right)\ll \text{1}$$

$$\text{rA}{\text{i}}_{\text{NxZo}}\text{=}\left(\text{A}{\text{i}}_{\text{NxZo}}\right)\text{/}\left(\text{A}{\text{i}}_{\text{NxAf}}\right)\text{≪1}$$

$$\text{rA}{\text{i}}_{\text{NxLt}}\text{=}\left(\text{A}{\text{i}}_{\text{NxLt}}\right)\text{/}\left(\text{A}{\text{i}}_{\text{NxAf}}\right)\text{<1}$$

$$\text{rA}{\text{i}}_{\text{NxVm}}\text{=}\left(\text{A}{\text{i}}_{\text{NxVm}}\right)\text{/}\left(\text{A}{\text{i}}_{\text{NxAf}}\right)\ll \text{1}$$

**Figure 3:**
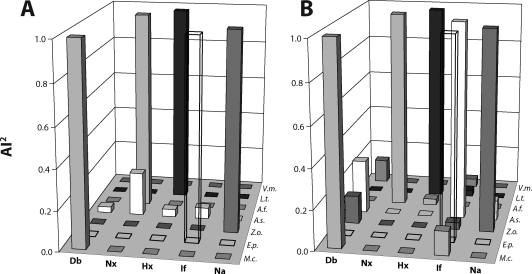
Product of the relative association indices (rAi's) for each plant-biomedical term association. Each specific bar from Figure 1 was multiplied by its corresponding bar in Figure 2 to yield the highly specific rAi^2^ value (indicated as ‘AI^2^’ on the y-axes). (**A**) graph based on Medline PubMed database; (**B**) graph based on the much larger and less-specific world-wide-web (www) database.

An additional calculation is performed to emphasize the relative strengths of rAi values by squaring the two corresponding values (one based on Figure 1 values, and the other on Figure 2 values) to yield the most specific association parameter, rAi^2^. These rAi^2^ values are graphed in Figure 3 and their significance is further discussed in the next section. As an example, the rAi^2^ value for the Db-*M.c.* relation using the values from Figure 1 and 2 was calculated as follows (result is graphed in Figure 3A): rAi^2^ = (rAi_McDb_) (rAi_DbMc_) = (1) (1) = 1. As an additional example, the rAi^2^ value for the Nx-*A.s.* relation using the values from Figure 1 and 2 was calculated as follows (result is graphed in Figure 3A): rAi^2^ = (rAi_AsNx_) (rAi_NxAs_) = (1) (0.2) = 0.2

## Results and discussion

In terms of the biomedical-specific database (Medline), Figure 1 shows the relative relation (relative association index, rAi) of each of the seven plants with all five biomedical parameters; whereas Figure 2 shows the rAi of each of the five biomedical parameters with all seven of the plants examined. Thus, for each plant-biomedical parameter relation, there are two possible rAi's, one based on the most active plant for a given biomedical parameter, and the other based on the most affected biomedical parameter for a given plant. This paper proposes a novel combination of these two rAi's to yield a highly specific indicator, rAi^2^, for a given plant-biomedical parameter relation. To allow comparisons of data from two large databases, we ultimately calculated rAi^2^ of a given plant-biomedical parameter relation for both the biomedical-specific database (Medline, Figure 3A) and the less-specific global internet database (www, Figure 3B).

In terms of toxicity, the results indicate that *Aristolochia (A.f.)* exhibits a major relation with nephrotoxicity; this was observed both through a selection process based on plant activities (Figure 1) and based on biomedical parameters (Figure 2). This major relation is expected based on the evidence that *Aristolochia* phytochemicals are causally involved in such toxicity (see Introduction for references). The results do not show evidence for *Aristolochia* hepatotoxicity; but instead, the results of Figures 1 and 2 identify and quantify the evidence base for the hepatotoxicity of *Larrea (L.t.)*. In the more stringent analysis (rAi^2^) represented by Figure 3, *Larrea-*hepatotoxicity and *Aristolochia-*nerphrotoxicity are the only two major plant-toxicity associations (note a lack of *Larrea*-nephrotoxicity association). The two plant-toxicity associations are identified in both the Medline database (Figure 3A) and the very large, less-specific, www (Figure 3B) database. These two databases are compared further below.

Two opposite effects can contribute to a toxicity relation for a given plant, one as a cause of the toxicity and the other as a protective (or anti-toxic) effect. *Allium* has a strong relation with four of the biomedical parameters, including Nx and Hx, in terms of rAi (Figure 1); but as shown in Figure 3, the strength of the associations (especially for Hx and Nx) is diminished with the more stringent rAi^2^. It should be noted in this context that there is a large database of reported therapeutic effects of *Allium* for many biomedical parameters including protection of kidney and liver from toxicity caused by other compounds and plants. Similarly, of the five biomedical effects analyzed in this study, the major reported activity for *Echinacea (E.p.)* is anti-inflammatory (cf. results of current study in Figures 1–3), and the major reported activity for *Zingiber (Z.o.)* is anti-nausea (cf. results of current study in Figures 1–3).

The data in Figure 3 also verify the diabetes-*Momordica* relation that was included as an additional positive control (see Introduction). As a negative control, *Vaccinium (V.m.)* has a wide range of potential effects, and the evidence in the databases is not dominated by any one of the five biomedical parameters used in the current study; Figure 3 shown no major biomedical parameter relations for *Vaccinium*. Moreover, the *Allium* (anti-)inflammation relation that is prominent in the global www database (Figure 3B) is not confirmed in Medline (Figure 3A), a database with a more stringent selection for scientific evidence in relation to biomedical applications. Medline is likely to provide a better estimate of existing evidence for toxic or therapeutic effects, and also for biochemical or clinical research activity related to the health parameters of a given plant or its constituent phytochemicals. The internet (www), as a general database, is influence by a relatively large number of factors that are less related to scientific evidence, *e.g.*, proposed therapeutic applications by product vendors or applications based on traditional uses.

The phytomedicinal informatics procedure presented in this report is more generally applicable to toxicological research. Large databases that include toxicological effects of plants, plant extracts, or specific phytochemicals may be searched; and the evidence for an association of the plants or compounds to the specific toxicity target sites (*e.g.*, organs, tissues, cells) or toxicity processes (*e.g.*, cell transport, signaling, gene expression, DNA damage) can be quantified. The current study introduces a novel rAi^2^ parameter that incorporates information from large databases to identify and quantify relative toxic effects (and other effects for comparison) of a group of medicinal plants, and relative toxicity of a given medicinal plant. The example provided by the study identifies two plants with major evidence for hepato- or nephrotoxic effects, and quantifies the relative evidence for all of the seven plants analyzed. Moreover, because these databases are dynamic, it is important to emphasize the need to reevaluate the associative parameters such as rAi^2^ on a regular basis to incorporate the results of novel studies.
